# IR‐Laser Ablation of Potassium Cyanide: A Surprisingly Simple Route to Polynitrogen and Polycarbon Species

**DOI:** 10.1002/chem.201905103

**Published:** 2020-01-23

**Authors:** Frenio A. Redeker, Helmut Beckers, Sebastian Riedel

**Affiliations:** ^1^ Institut für Chemie und Biochemie Anorganische Chemie Freie Universität Berlin Fabeckstrasse 34/36 14195 Berlin Germany

**Keywords:** laser ablation, matrix-isolation spectroscopy, polycarbon compounds, polynitrogen compounds, quantum-chemical calculations

## Abstract

Pulsed laser irradiation of solid potassium cyanide (KCN) produces, besides free nitrogen and carbon atoms, the molecular species KN and KC which are potential candidates for interstellar species of potassium. Additionally, N_3_, N_3_^−^, KN_3_, C_3_, C_3_^−^, and KC_3_ are produced and isolated in solid noble gases as well as in solid N_2_. Molecular potassium nitrene (KN) reacts with dinitrogen in neon and argon matrices after photochemical excitation (*λ*=470 nm) forming molecular end‐on (*C*_∞v_) and side‐on (*C*_2v_) potassium azide isomers. The side‐on isomer (*C*_2v_) is thermodynamically favored at the CCSD(T)/ma‐def2‐TZVP level of theory. It can be obtained from the end‐on isomer by UV‐irradiation (*λ*=273 nm).

## Graphical abstract

**Pulsed laser irradiation** of solid potassium cyanide (KCN) produces the molecular species KN and KC, which are potential candidates for interstellar species of potassium. Molecular potassium nitrene (KN) reacts with dinitrogen in neon and argon matrices after photochemical excitation forming two molecular potassium azide isomers, end‐on and side‐on KN_3_.

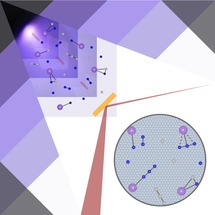

Potassium cyanide is one of the metal cyanide species detected in proximity to a star in space^1^ and KC, KN, and KO are supposed to be potential candidates for further interstellar species of potassium.^2^ While KC and KO were characterized by their rotational spectra,^3^ to our knowledge no spectroscopic gas‐phase study has yet been published for KN. However, potassium nitrene has been predicted to be stable with a bond dissociation energy of 81 kJ mol^−1^ at the MRCI(+Q)/triple‐ζ level of theory.^2^

**Figure 1:**
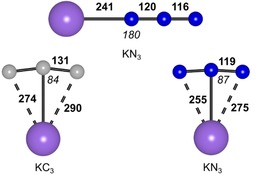
Structures of the KC_3_, end‐on KN_3_, and side‐on KN_3_ ion pairs computed at the CCSD(T)/ma‐def2‐TZVP level of theory. Bold and italic numbers indicate bond lengths [pm] and bond angles [°], respectively.

Pulsed IR‐laser ablation of solid salts was recently shown to be a particularly suitable method to produce and characterize anionic complexes. Recent examples are the homoatomic anions Cl_3_^−^ and F_3_^−^, which were produced by co‐deposition of IR‐laser ablated potassium halide, KX ( X=Cl and F, respectively), with gaseous X_2_/noble gas mixtures at cryogenic temperatures.^4^ Two further homoatomic anions, which are of particular interest for the present work, are the C_3_^−^ and N_3_^−^ anions. The first one was assigned by Szczepanski et al. to a band at 1721.8 cm^−1^ after isolation and electron bombardment of laser ablated graphite in a solid argon matrix.^5^ The free N_3_^−^ anion was associated with an IR band at 2003.5 cm^−1^ by Michl and co‐workers, which was observed in pure N_2_ matrices after atom/ion bombardment.^6^ It was later also detected by Andrews and co‐workers after co‐deposition of laser ablated Ga, In, and Tl atoms in solid nitrogen,^7^ and in argon matrices, where this band appeared at 1991.9 cm^−1^.^8^

As a logical extension of our former work^4^ we present in this study results obtained by laser ablation of the ternary system potassium cyanide (KCN). Matrix‐isolation IR‐spectra of thermally evaporated sodium and potassium cyanide were reported previously by Ismail, Hauge, and Margrave (IHM).^9^ The IR spectra obtained by us from IR‐laser ablation of potassium cyanide deposited in solid argon are in the spectral region above 2000 cm^−1^ are very similar to those described by IHM. We find KNCO and CO as the main impurities in our spectra due to the high temperature reaction of KCN with CO_2_ formed by laser ablation of CO_3_^−^ impurities. The CN stretching region of the IR spectra of laser ablated KCN isolated in neon and argon is shown in Figures S1 and S2 in the Supporting Information. Based on the work by IHM, the observed bands at 2048.7, 2059.3/2061.3, and 2079.6 cm^−1^ in argon and 2047.0, 2061.2, and 2075.7 cm^−1^ in neon are assigned to monomeric, oligomeric, and polymeric potassium cyanide, respectively. In contrast to the thermal evaporation, which essentially results in isolated monomeric and oligomeric ion pairs, laser ablation allows for the preparation of free anions, for example, laser ablation of alkali halides (MX, X=Cl, F) allowed us to study not only isolated MX ion pairs in solid noble gas matrices, but also to isolate free anions such as the free X_3_^−^ ions in the presence of X_2_.^4^ It was, therefore, rather surprising to find that laser ablation of KCN does not produce free CN^−^ ions, which have a reported band at 2053.1 cm^−1^ in solid neon^10^ (cf. Figure S1, Supporting Information).

In IR spectra obtained from laser ablated potassium cyanide trapped in solid argon at 12 K we found two new bands in a region at 1722.0 and 1712.5 cm^−1^ where we do not expect any CN stretching bands (Figure 2). Both bands reveal a large ^13^C shift of −66 cm^−1^ in experiments using K^13^CN, and when KCN was replaced by NaCN it became evident that the 1722.0 cm^−1^ band is metal independent, whereas the 1712.5 cm^−1^ band is not. The metal‐independent band is close to the band at 1721.8 cm^−1^ previously assigned by Szczepanski et al. to the antisymmetric stretch (*ν*_3_) of the linear C_3_^−^ anion.^5^ Here we confirm the assignment of this band based on its metal independency and its ^12/13^C isotope pattern obtained after pulsed laser deposition of a 1:1 mixture of K^12^CN and K^13^CN in argon (Figure S6, Supporting information). The 1:1 isotopic mixture yielded six ^12/13^C_3_^−^ isotopologues. The band positions of all C_3_^−^ isotopologues are displayed in Table 1.

**Figure 2:**
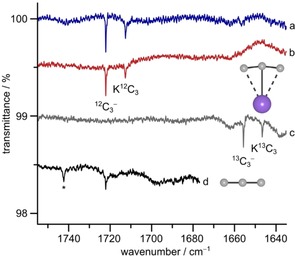
IR spectra recorded after deposition of laser ablated a) K^12^C^14^N, b) K^12^C^15^N, c) K^13^C^14^N, and d) Na^12^C^14^N in excess argon at 12 K. * Unknown species.

**Table 1:** Band positions [cm^−1^] of the antisymmetric C_3_^−^ stretching vibration (*ν*_3_) of the isotopologues of free C_3_^−^ (*D*_∞h_) and T‐shaped KC_3_ (*C*_2v_).^[a]^

Species	121212	131212	131213	121312	131312	131313
C_3_^−^ (this work)^[b]^	1722.0	1711.5 (−10.5)	1700.2 (−21.8)	1678.1 (−43.9)	1667.3 (−54.7)	1655.6 (−66.4)
C_3_^−^ (Ref. 5b)^[b]^	1721.8	1711.2 (−10.6)	1699.9 (−21.9)	1677.7 (−44.1)	1667.1 (−54.7)	1655.5 (−66.3)
KC_3_ (this work)^[b]^	1712.5	1701.9 (−10.6)	1691.1^[d]^ (−21.4)	1668.8^[d]^ (−43.7)	1657.2^[d]^ (−55.3)	1646.5 (−66.0)
KC_3_ (this work)^[c]^	1727.1	1716.4 (−10.7)	1704.8 (−22.3)	1682.3 (−44.8)	1671.3 (−55.8)	1659.4 (−67.7)

[a] Isotopic shifts with respect to the main isotopologue are given in parentheses. The isotopologues of C_3_^−^ are abbreviated by the masses of their carbon isotopes: 131212 is equivalent to K^13^C^12^C^12^C. Note that 131212 and 121313 are equivalent to 121213 and 131312, respectively. [b] Solid argon. [c] Calculated harmonic wavenumbers (CCSD(T)/ma‐def2‐TZVP). [d] Tentatively assigned band with low intensity (Figure S6, Supporting Information).

The metal‐dependent band at 1712.5 cm^−1^ in Figure 2 has almost the same ^13^C isotopic shift as the free C_3_^−^ anion. It is assigned to the *ν*_3_ stretch of the corresponding ion pair KC_3_ (*C*_2v_) which has been predicted to be the most stable potassium doped carbon cluster KC_*n*_ (*n*=1–10) by a recent DFT study.^11^ Our quantum‐chemical calculations at the CCSD(T)/ma‐def2‐TZVP level also support this assignment (Table 1, Figure 1). The full isotope pattern of KC_3_ is not as nicely resolved as for C_3_^−^ due to lower yields (Figure S6, Supporting Information), so that only three of its isotopologues can be confidently assigned. The band at 1742.3 cm^−1^ observed after laser ablation of natural NaCN (Figure 2 d) immediately suggests an assignment to NaC_3_ since the *ν*_3_(C_3_^−^) stretch of MC_3_ is intuitively expected to shift to higher wavenumbers for lighter alkali metals. However, CCSD(T) calculations predict the *ν*_3_(C_3_^−^) of NaC_3_ 16 cm^−1^ lower than for KC_3_. Therefore, this band must yet remain unassigned.

Upon irradiation of the neon matrices at 6 K with blue LED‐light (*λ*=470 nm) two new bands at 2070.8 and 1998.4 cm^−1^ appeared (Figure 3 a). Subsequent irradiation with a *λ*=273 nm led to conversion of the 2070.8 cm^−1^ into the 1998.4 cm^−1^ band, indicating that the two bands correspond to two different molecular species. In solid argon at 12 K these bands shifted to 2057.0 and 1988.9 cm^−1^ (Table 2) and their intensity increased by addition of small amounts of N_2_ to the matrix gas. While no ^12/13^C isotope shifts were observed in the experiments performed with K^13^CN, both bands revealed large ^14/15^N isotope shifts of −68.3 (2070.8 cm^−1^) and −65.8 cm^−1^ (1998.4 cm^−1^), respectively, in experiments using ^15^N isotopically enriched KC^15^N and ^15^N_2_ doped neon (Figure 3 b). These large isotope shifts are strong evidence that only nitrogen atoms are involved in the corresponding vibrational modes.

**Figure 3:**
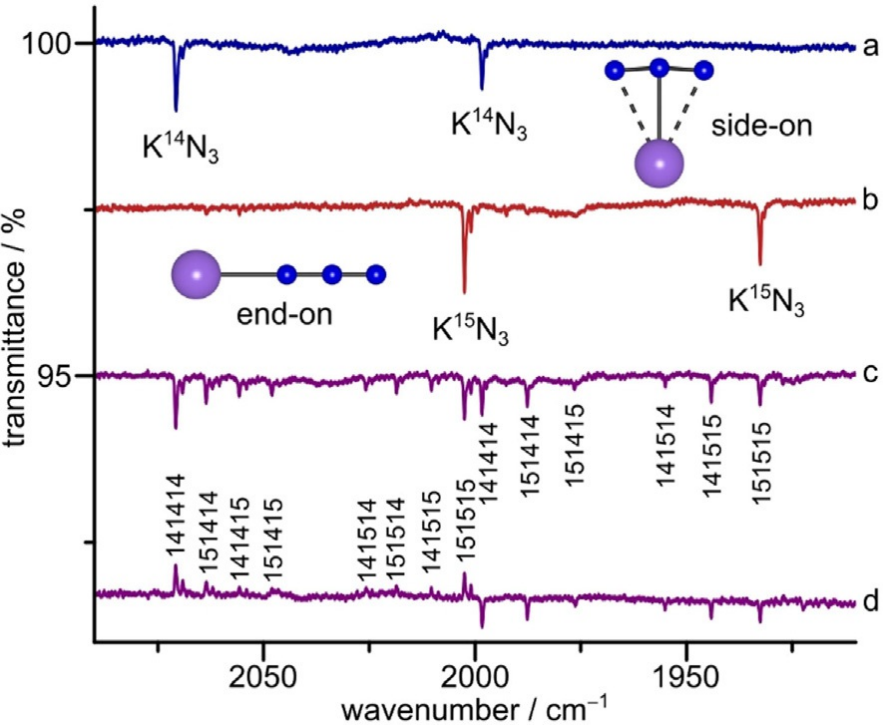
IR spectra recorded after co‐deposition of laser ablated KCN with N_2_ in solid Ne at 6 K. Difference spectra a) after deposition of K^12^C^14^N with ^14^N_2_ (0.5 %) in Ne and photolysis (*λ*=470 nm), b) after deposition of K^12^C^15^N with ^15^N_2_ (0.5 %) in Ne and photolysis (*λ*=470 nm), c) after deposition of K^12^C^14^N/K^12^C^15^N (1:1) with ^14^N_2_/^15^N_2_ (1:1, 0.2 %) in Ne and photolysis with *λ*=470 and d) after photolysis of c) with *λ*=273 nm. Bands pointing upwards indicate depletion and bands pointing downwards formation of the corresponding species.

**Table 2:** Band positions [cm^−1^] of IR active N_3_ stretches of the end‐on (*C*_∞v_) and side‐on (*C*_2v_) potassium azide isotopologues.^[a]^

*C*_∞v_	141414	151414	141415	151415	141514	151514	141515	151515
*ν_3_* (calcd)^[b]^	2124.5	2116.7 (−7.8)	2108.7 (−15.8)	2100.3 (−24.2)	2077.1 (−47.4)	2069.3 (−55.2)	2060.9 (−63.6)	2052.4 (−72.1)
*ν_3_* (solid Ne)	2070.8	2063.6 (−7.2)	2055.8 (−15.0)	2048.0 (−22.8)	2025.8 (−45.0)	2018.6 (−52.2)	2010.3 (−60.5)	2002.5 (−68.3)
*ν_3_* (solid Ar)	2057.0	2049.3 (−7.7)	n.o.	n.o.	n.o.	n.o.	n.o.	1989.2 (−67.8)
*ν_3_* (solid N_2_)	2048.5	2039.9 (−8.6)	2035.0 (−13.5)	2026.2 (−22.3)	2003.4 (−45.1)	1995.1 (−53.4)	1989.9(‐58.6)	1980.9 (−67.6)
*ν_1_* (calcd)^[b]^	1357.5	1330.9 (−26.6)	1338.0 (−19.5)	1311.7 (−45.8)	1357.3 (−0.2)	1330.5 (−27.0)	1337.9 (−19.6)	1311.5 (−46.0)
*ν_1_* (solid Ne)	1344.6	n.o.	n.o.	n.o.	n.o.	n.o.	n.o.	1299.6 (−45.0)

*C*_2v_	141414		151414	151415		141514	151514	151515
*ν*_3_ (calcd)^[b]^	2038.8		2027.5 (−11.3)	2015.7 (−23.1)		1993.4 (−45.4)	1981.8 (−57.0)	1969.7 (−69.1)
*ν*_3_ (solid Ne)	1998.4		1987.6 (−10.8)	1976.5 (−21.9)		1955.1 (−43.3)	1944.2 (−54.2)	1932.6 (−65.8)
*ν*_3_ (solid Ar)	1988.9		1978.3 (−10.6)	n.o.		1945.9 (−43.0)	1935.2 (−53.7)	1923.5 (−65.4)
*ν*_3_ (solid N_2_)	2006.5		1996.1 (−10.4)	1984.5 (−22.0)		1962.9 (−43.6)	1951.6 (−54.9)	1940.5 (−66.0)

[a] Isotopic shifts with respect to the main isotopologue are given in parentheses. The isotopologues of N_3_^−^ are abbreviated by the masses of their nitrogen isotopes: 151414 is equivalent to K^15^N^14^N^14^N. In case of the linear isomer the potassium ion is sitting to the left of the number series. In case of the T‐shaped isomer 151414 and 141515 are equivalent to 141415 and 151514, respectively. Bands that were too weak for a clear assignment are indicated with n.o. (not observed). [b] Calculated harmonic wavenumbers (CCSD(T)/ma‐def2‐TZVP).

In experiments using a mixture of KC^14^N/KC^15^N (1:1) as well as neon gas doped with a 1:1 ^14^N_2_/^15^N_2_ mixture (Figure 3 c), the higher band split into eight and the lower into six bands. In analogy with C_3_^−^ and KC_3_, a pattern of six isotopologues would be expected for the free N_3_^−^ anion or side‐on KN_3_ (*C*_2v_, Figure 1). The pattern of eight bands is indicative of three chemically inequivalent nitrogen atoms as in molecular end‐on KNNN (*C*_∞v_, Figure 1). Thus the higher‐frequency band at 2070.8 cm^−1^ is assigned to the *ν*_3_(N_3_^−^) stretch of molecular end‐on KN_3_ which is in good agreement with the calculated band position (2124.5 cm^−1^, CCSD(T)/ma‐def2‐TZVP), as well as with the predicted ^14/15^N isotopic shifts (Table 2). The lower‐frequency band at 1998.4 cm^−1^ is in good agreement with the computed *ν*_3_ band position of side‐on KN_3_ (2038.8 cm^−1^, CCSD(T)/ma‐def2‐TZVP) and close to the reported *ν*_3_ stretch of the free N_3_^−^ anion in solid nitrogen at 2003.5 cm^−1^.6a, 7 The conversion of end‐on KN_3_ into the carrier of the 1998.4 cm^−1^ band during UV irradiation (*λ*=273 nm, Figure 3 d) supports its assignment to side‐on KN_3_ rather than to free N_3_^−^. The symmetric stretch of side‐on KN_3_ (*ν*_1_) has almost no IR intensity and, therefore, could not be observed in our experiments, while the corresponding mode of end‐on KN_3_ has some IR intensity and could be observed for end‐on K^14^N_3_ and K^15^N_3_ in solid neon (Table 2, Figure S9, Supporting Information).

In solid nitrogen matrices no irradiation was needed to produce KN_3_. After deposition the ^14^N spectra showed a strong band at 2048.5 with a site at 2049.3 cm^−1^ for end‐on K^14^N_3_ and the broad band of free ^14^N_3_^−^ as described by Michl and Andrews at 2003.5 with a site at 2005.6 cm^−1^. Upon annealing to 25 K the band at 2048.5 cm^−1^ increased drastically while the N_3_^−^ band decreased so that another band at 2006.5 cm^−1^ became visible. The 2006.5 cm^−1^ band has not been described by Michl or Andrews and is assigned to the antisymmetric azide stretch (*ν*_3_) of side‐on KN_3_ in solid nitrogen. In the mixed isotope experiment where laser ablated KC^14^N/KC^15^N (1:1) was co‐deposited with pure ^14^N_2_/^15^N_2_ (1:1) at 12 K (Figure S7, Supporting Information), the band at 2048.5 cm^−1^ split into an octet, whereas the band at 2006.5 cm^−1^ split into a sextet, as expected for end‐on and side‐on KN_3_ (Figure S7b, Supporting Information). Upon irradiation with UV light (*λ*=273 nm) the free N_3_^−^ band was depleted, while the 2048.5 and 2006.5 cm^−1^ bands were not (Figure S7c, Supporting Information). All calculated and observed experimental band positions of the antisymmetric N_3_^−^‐stretches of side‐on and end‐on KN_3_ in Ne, Ar, and N_2_ are displayed in Table 2.

Calculations at the CCSD(T)/ma‐def2‐TZVP level of theory predict that side‐on KN_3_ is 3.5 kJ mol^−1^ lower in energy than end‐on KN_3_. The barrier for the rearrangement from end‐on to side‐on obtained from a relaxed surface scan along the K−N−N bond angle (Figure 4) is predicted to be 12.5 kJ mol^−1^ which appears to be high enough that the rearrangement could not be observed by annealing in any experiments. In neon, however, there seems to be a photochemical equilibrium between the end‐on and the side‐on form: Immediately after formation of the two isomers by irradiation with blue light (*λ*=470 nm) the ratio end‐on/side‐on was about 3/2 whereas after irradiation with UV light (*λ*=273 nm) it changed to 2/3. While this interconversion was only observed in neon, in argon, the side‐on KN_3_ is the main product after *λ*=470 nm photolysis.

**Figure 4:**
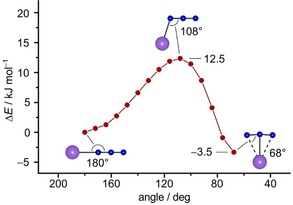
Relaxed surface scan of the potassium azide bond angle at the CCSD(T)/ma‐def2‐TZVP level of theory.

In principle, three reaction mechanisms can be considered for the formation of molecular KN_3_ in the experiments described above: i) recombination of K^+^ and N_3_^−^ ions, ii) reaction of potassium atoms and N_3_ radicals, and iii) reaction of potassium nitrene (KN) and N_2_ molecules [Eq. (1)]. In the neon and argon experiments, however, the former two reactions can be ruled out since neither N_3_^−^ anions nor N_3_ radicals were observed in these matrices. KN is calculated to have a triplet ground state (MRCI(+Q)),^2^ while the reaction of KN+N_2_ (1) is assumed to proceed with excited singlet KN molecules after photoexcitation and intersystem crossing (ISC) to their lowest singlet state. Note that we have not observed the vibrational band of KN (324.4 cm^−1^)^2^ in our experiments, because this band is expected to be rather weak and beyond the range of our MCT detector. Nevertheless, KN should be present as a key intermediate for the formation of KN_3_.

(1) $$\mathrm{KN}+N{}_{2}\overset{hv}{\underset{}{\to }}\mathrm{KN}{}_{3}$$

In solid nitrogen, large amounts of end‐on KN_3_ and only small amounts of side‐on KN_3_ were observed after annealing to 25 K. At the same time high amounts of free N_3_^−^ were consumed during this process, suggesting that KN_3_ might also be formed by a recombination of K^+^ and N_3_^−^ ions in solid nitrogen. This observation explains the high abundance of end‐on compared to side‐on KN_3_ in solid nitrogen: The partial negative charges on the two terminal nitrogen atoms in N_3_^−^ favor an electrostatic recombination of K^+^ and N_3_^−^ and the formation of the thermodynamically less stable end‐on KN_3_.

Figure 5 shows the ^14/15^N isotopic IR band patterns obtained after co‐deposition of laser ablated KC^14^N with ^15^N_2_ doped neon (a) and of KC^15^N with ^14^N_2_ doped neon (b) after irradiation with blue light (*λ*=470 nm). Obviously, spectra Figure 5 a and b are complementary. Due to the purity of 98 atom % ^15^N of the KC^15^N used for the experiment, traces of the K^14^N^14^N^14^N isotopologue can be observed in Figure 5 b. The high yield of K^15^N_3_ in Figure 5 b indicates that ^15^N_2_ is formed in substantial amounts by recombination of ^15^N atoms from laser ablated KC^15^N. The absence of any (^15^N_2_^14^N) isotopologues in Figure 5 b and of (^14^N_2_^15^N), and K^15^N^15^N^15^N isotopologues in Figure 5 a implies the absence of a reaction between K^15^N and ^14^N_2_ producing K^14^N and ^14^N^15^N or vice verca. On the other hand, K^14^N can react with ^15^N_2_ not only to form the addition product K^14^N^15^N^15^N but also to form K^15^N^14^N^15^N and K^15^N^15^N^14^N. This observation may indicate a K(*η* ^3^‐N_3_) ion‐pair‐intermediate or transition state involving a cyclic *η* ^3^‐N_3_^−^ ring in the course of the photoinduced reaction that finally rearranges to one of the three possible mixed isotopologues according to Equation (2). Such a transient cyclic N_3_^−^ has already been postulated by Michl and co‐workers.6b

**Figure 5:**
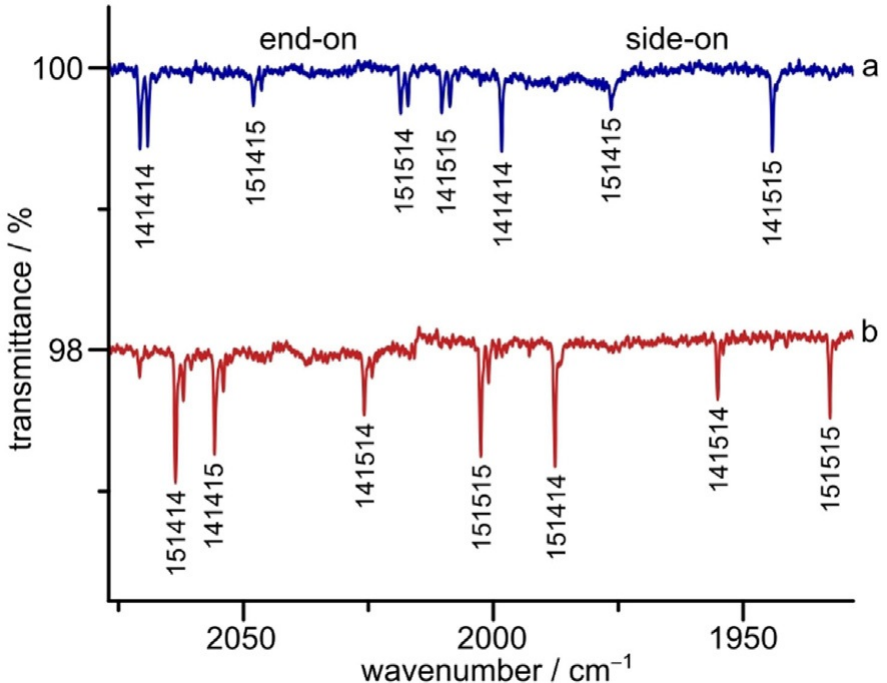
IR spectra recorded after co‐deposition of laser ablated KCN with N_2_ in solid Ne at 6 K. Difference spectra after photolysis (*λ*=470 nm, 10 min) of a) K^12^C^14^N with ^15^N_2_ (0.2 %), b) K^12^C^15^N with traces of ^14^N_2_. Bands pointing downwards indicate formation of the corresponding species.

In addition to the free C_3_^−^ and N_3_^−^ anions and the ion pairs KC_3_ and KN_3_ the IR spectra of the deposits obtained from IR laser ablation of potassium cyanide also showed bands which can be attributed to well‐known free radicals such as the C_3_,^12^ N_3_,6a CN,^10^ NCN,^13^ and CNN^14^ radicals (for further details and a discussion of the reaction mechanisms see the Supporting Information).

(2) $$K{}^{14}N+{}^{15}N{}_{2}\overset{hv}{\underset{}{\to }}K\left(\eta {}^{3}\text{-}{}^{14}N{}^{15}N{}_{2}\right){}^{*}\to K{}^{14}N{}^{15}N{}^{15}N\;\mathrm{or}\;K{}^{15}N{}^{15}N{}^{14}N\;\mathrm{or}\;K{}^{15}N{}^{14}N{}^{15}N$$

In the present paper we have shown that IR laser ablation of potassium cyanide leads to a complex mixture of polynitrogen and polycarbon species. In this study, molecular KN_3_ and KC_3_ are reported for the first time. To the best of our knowledge, previous IR spectroscopic studies on potassium azide were performed only on crystalline KN_3_,^15^ while several studies were reported on molecular group 2,^16^ group 13,^7^, ^8^ and transition‐metal azides.^17^ The ion pair KN_3_ exists in an end‐on and a side‐on form, which are separated by a barrier of about 12 kJ mol^−1^ at coupled‐cluster level. Molecular potassium nitrene (KN) is assumed to be a key intermediate produced by IR‐laser ablation of potassium cyanide (KCN), that reacts photochemically with dinitrogen to molecular potassium azide (KN_3_). Laser ablation of KCN could therefore be a suitable route for the gas‐phase generation and spectroscopic detection of the elusive KN molecule, which is of interest as a potential interstellar molecule. Awareness of the photochemical reaction of KN and N_2_ might also enable a better understanding of mechanisms involved in processes of photochemical nitrogen fixation.

## Conflict of interest

The authors declare no conflict of interest.

## Supporting information

As a service to our authors and readers, this journal provides supporting information supplied by the authors. Such materials are peer reviewed and may be re‐organized for online delivery, but are not copy‐edited or typeset. Technical support issues arising from supporting information (other than missing files) should be addressed to the authors.

Supplementary
