# Supplementary material for: IR‐Laser Ablation of Potassium Cyanide: A Surprisingly Simple Route to Polynitrogen and Polycarbon Species
Source: Chemistry. 2020 Jan 23;26(8):1763–7. doi: 10.1002/chem.201905103 (PMC7027886; doi:10.1002/chem.201905103)
Supplement: Supplementary file 1 — Supplementary [file CHEM-26-1763-s001.pdf]

CHEMISTRY
A European Journal

Supporting Information

IR-Laser Ablation of Potassium Cyanide: A Surprisingly Simple Route to Polynitrogen and Polycarbon Species

Frenio A. Redeker, Helmut Beckers, and Sebastian Riedel*[a]

chem_201905103_sm_miscellaneous_information.pdf

S1. Experimental and computational details 1

S2. Suggested reaction mechanisms leading to polycarbon, polynitrogen, and mixed species during pulsed IR laser ablation of potassium cyanide and during deposition 2

S3. Additional spectra 5

S4. Tables of additional experimental IR bands 9

S5. Calculated structures and harmonic frequencies 10

S6. References 11

S1. Experimental and computational details

Matrix-isolation experiments were carried out in a custom built vacuum chamber equipped with a rotatable target holder, in which a vacuum of < $6 \cdot 10^{-6}$ mbar was maintained over the whole period of an experiment. A gold plated copper block was used as matrix support and was cooled to 12 K (Ar, N_2) and 6 K (Ne), respectively, using a cold-head with a helium compressor unit. IR spectra were recorded in reflection using a Bruker Vertex 80v vacuum FTIR spectrometer equipped with a transfer optic, a KBr beam splitter, and a liquid nitrogen cooled MCT detector in a spectral range of 4000-400 cm^{-1} with a resolution of 0.2 cm^{-1}. A pulsed Nd:YAG laser (1064 nm) focused onto the rotating target by a plano-convex lens was used for laser ablation at a pulse rate of 1 Hz, a pulse length of 3-7 ns and a pulse energy of ~50 mJ. Matrices were deposited at a rate of 1 mbar L min^{-1} over time periods of 1 h (Ar, N_2) and 1.5 h (Ne). For irradiation of matrices, water cooled monochromatic LEDs in the visible (λ = 730, 590, 528, and 470 nm, P = 8000 mW) and UV (λ = 273 nm, P = 70 mW) range were used. Powdery potassium cyanide (KCN, purity > 98 %), and isotopically enriched samples ($K^{13}CN$: 99 atom % ^{13}C, $KC^{15}N$: 98 atom % ^{15}N) were pressed into a cylindrical shape using a hydraulic lab press. Mixtures of natural and isotopically enriched samples were thoroughly ground and mixed beforehand.

All quantum chemical calculations were carried out using the Orca 4.0.1 program package.[1] Molecular structures were first optimized at the DFT level using the BP86 functional[2] using dispersion correction (D3BJ)[3] and minimally augmented Karlsruhe basis sets (ma-def2-TZVP)[4] for all atoms. Minima found thereby were further optimized at the CCSD(T)/ma-def2-TZVP level of theory. Harmonic vibrational frequencies and reaction enthalpies were also computed at the CCSD(T)/ma-def2-TZVP level. Calculated data can be found in section 3 of the supporting information.

S2. Suggested reaction mechanisms leading to polycarbon, polynitrogen, and mixed species during pulsed IR laser ablation of potassium cyanide and during deposition

In addition to the free anions and ion pairs (see main text) the IR spectra of deposits obtained from IR laser ablation of potassium cyanide also showed a prominent band which can be assigned to well-known radicals such as the C_3 radical[5] in solid Ne 1936.6 cm^{-1}, Figure S1) and argon (1939.2 cm^{-1}, Figure S2) and high amounts of free N_3 radicals[6] in pure nitrogen matrices (ν_3, 1657.9 cm^{-1}, Figure S3). The observation of the C_3 radical by laser ablation of KCN suggests that free carbon atoms are formed probably by reaction (1) or (2) or both during the laser ablation.

$$KCN \xrightarrow{h\nu} K + C + N \quad (1)$$

$$KCN \xrightarrow{h\nu} KN + C \quad (2)$$

The free carbon atoms can then recombine in the gas phase and/or during deposition to successively yield C_3 by reactions (3) and (4). Note, reaction (3) is known to proceed at a fairly high rate at room temperature in the gas phase[7] and it has been shown that C_3 is the predominant species in hot carbon vapor[8].

$$C + C \longrightarrow C_2 \quad (3)$$

$$C + C_2 \longrightarrow C_3 \quad (4)$$

The high amounts of free N_3 radicals detected after matrix-isolation of laser-ablated KCN in pure nitrogen matrices, indicates that also N atoms are formed during laser ablation by reactions (1) and/or (5).

$$KCN \xrightarrow{h\nu} KC + N \quad (5)$$

After co-deposition of a 1:1 mixture of $KC^{14}N$ and $KC^{15}N$ with a 1:1 mixture of $^{14}N_2$ and $^{15}N_2$ at 12 K and annealing to 25 K, we observed the same six bands for these N_3 isotopologues (Figure S3, Table S3). An interesting feature is the observed intensity ratio of about 20:20:1:1:20:20. The fact that ν_3 of $^{14}N^{14}N^{14}N$, $^{14}N^{14}N^{15}N$, $^{15}N^{15}N^{14}N$, and $^{15}N^{15}N^{15}N$ all have about the same intensity suggests a reaction of $^{14/15}N$ atoms with unscrambled $^{14}N_2$ or $^{15}N_2$ molecules (Eq. 7). The much lower intensities of $^{15}N^{14}N^{15}N$ and $^{14}N^{15}N^{14}N$ suggest that no insertion of N atoms into N_2 occurred and that these two isotopologues are obtained from a reaction of $^{14/15}N$ atoms with $^{14}N^{15}N$ molecules formed by reaction (6). The recombination reaction (6) in the gas phase is believed to be a three-body reaction $N + N + X \rightarrow N_2 + X$,[9] where X could be any inert species, here most likely N_2.

$$N + N \longrightarrow N_2 \quad (6)$$

$$N + N_2 \longrightarrow N_3 \quad (7)$$

Also traces of CN (2043.8 cm^{-1}) and NCN (ν_3, 1466.9 cm^{-1}) radicals (Figure S1 and S4) were observed when laser ablated KCN was co-deposited with neon at 6 K. The assignment of CN is based on a band which Forney *et al.* reported after co-deposition of Ne/HCN with excited

(microwave discharge) neon atoms.[10] The observation of only traces of CN radicals in our neon experiments proves that the release of CN radicals unlikely occurs during laser ablation of KCN.

The NCN radical in solid neon was previously detected by Jacox and Thompson who co-deposited Ne/NCCN with excited neon atoms under cryogenic conditions.[11] In argon neither CN nor NCN radicals were detected, while in pure nitrogen matrices NCN and CNN were formed in comparably high yields (Figure S5). The band positions of NCN (ν_3, 1478.4 cm^{-1}) and CNN (ν_1, 1253.1 cm^{-1}) in solid nitrogen are in good agreement with those of previous studies[12,13] and their high abundance is further evidence for the formation of carbon atoms during laser ablation of KCN by reactions (1) and/or (2): The formation of NCN and CNN in nitrogen can be explained by the reactions of carbon atoms with N_2 (Eq. 8 and 9).[13]

$$C + N_2 \longrightarrow NCN \quad (8)$$

$$C + N_2 \longrightarrow CNN \quad (9)$$

$$N + CN \not\longrightarrow NCN \quad (10)$$

In solid neon and argon, reactions (8) and (9) are unlikely, because N_2 is far less abundant. Reaction (10) can be ruled out for the formation of NCN, since the $^{14}NC^{15}N$ isotopologue was not observed in any experiments with 1:1 mixtures of $KC^{14}N$ and $KC^{15}N$. The band positions of the discussed radical species and some further products in solid Ne, Ar, and N_2 are displayed in Tables S1-S3.

As mentioned above, recombination of carbon atoms in the gas phase and/or during deposition to yield C_2 (Eq. 3) and C_3 (Eq. 4) is known to be very fast.[7,8] For the formation of the C_3^- anion, which also has been detected in the product mixture (see main text and Figure S6) one can assume an electron produced by the laser ablation process is captured by the C_3 radical. However, a more detailed picture can be deduced from the $^{13/14}C$ isotope pattern of the ν_3 stretch of the C_3^- anion, obtained from a 1:1 mixture of $K^{12}CN$ and $K^{13}CN$ in solid argon. In fact the intensity distribution of the observed pattern is 2:2:1:1:2:2 (Figure S6) rather than the 1:2:1:1:2:1 expected for the ν_3 stretch of a C_3^- anion with two equivalent carbon atoms. This unusual intensity pattern most likely indicates that initially dicarbon species ($^{12}C_2$ or $^{13}C_2$) are primarily ejected from the $K^{12}CN/K^{13}CN$ target (Eq. 3), which may further react with $^{12/13}C^-$ ions according to Eq. 12. From this result it can be assumed that a perfectly mixed target can only be obtained by dissolving and recrystallizing a 1:1 mixture of $K^{12}CN$ and $K^{13}CN$. The C^- anions may be obtained directly by laser ablation of KCN (Eq. 11) or from initially formed carbon atoms after electron capture. Potassium has a low ionization energy compared with C and N (IE = 4.34 (K), 11.26 (C), 14.53 (N) eV)[14] while carbon atoms have a higher electron affinity than nitrogen atoms (EA = 0.50 (K), 1.26 (C), −0.07 (N) eV)[15]. Thus, the most likely ionization that can occur during laser ablation is where potassium will carry the positive and carbon the negative charge (Eq. 11). The observation of both, the free C_3^- anion and the ion pair KC_3 suggests the occurrence of reactions (12) and (13), while KC is provided by reaction (5).

$$KCN \xrightarrow{h\nu} K^+ + C^- + N \quad (11)$$

$$C^- + C_2 \longrightarrow C_3^- \quad (12)$$

$$KC + C_2 \longrightarrow KC_3 \quad (13)$$

The fact that free N_3^- has not been observed in our argon and neon matrices can be rationalized by remembering the electron affinities of nitrogen atoms (EA = −0.07 eV)[15] and of dinitrogen (EA_{B3LYP} = −1.90 eV)[16]. The species which most likely captures an electron is the N_3 radical (EA = 2.70 eV)[17]. Laser ablation yields electrons that are trapped in the matrix and can be captured by N_3 to yield N_3^-. In pure N_2 all nitrogen atoms produced by laser ablation will exothermically react with the matrix host N_2 molecules to N_3 radicals. Thus, it is no surprise that in neon and argon, where N_3 radicals were not observed, N_3^- is not found, either.

Michl *et al.* and Andrews *et al.* observed, in addition to the ν_3 of free N_3^- stretching band at 2003.5 cm^{-1}, a band at 2077 cm^{-1} in pure nitrogen matrices.[6,18,19] This band, which was assigned to an ion-paired $X^+N_3^-$, was not observed in our experiments. In solid argon and nitrogen the antisymmetric stretch of end-on KN_3 (2057.0 and 2048.5 cm^{-1}, respectively) are close to the values for solid potassium azide[20] and for molecular end-on TlN_3 islolated in solid nitrogen[18], confirming the ionic character of the $K^+N_3^-$ interaction. In neon, the interaction with the matrix gas is weaker pushing the band to higher wavenumbers (2070.8 cm^{-1}) in the direction of more covalently bound end-on azide complexes like InN_3 and GaN_3[18].

In addition to KN_3, N_3^-, and N_3, two other polynitrogen species were detected in some experiments. The linear N_4^+ (ν_{as}, 2237.8 cm^{-1}), already described by Jacox as a product of N_2 co-deposited with excited Ne atoms from a microwave discharge,[21] was observed after codeposition of laser ablated KCN in nitrogen doped neon. Another, metal dependent band was observed at 1498 cm^{-1} in these experiments (Figure S8) and at 1575 cm^{-1} in pure nitrogen experiments. This band does not reveal a ^{13}C isotope shift and shows a triplet $^{14/15}N$ isotope pattern in experiments with $^{14}N_2$ + $^{15}N_2$ (1:1), indicating that its carrier contains two chemically equivalent N_2 units. The band was also produced by co-deposition of laser ablated potassium with pure nitrogen at 12 K. The associated species, however, remains so far unknown.

S3. Additional IR spectra

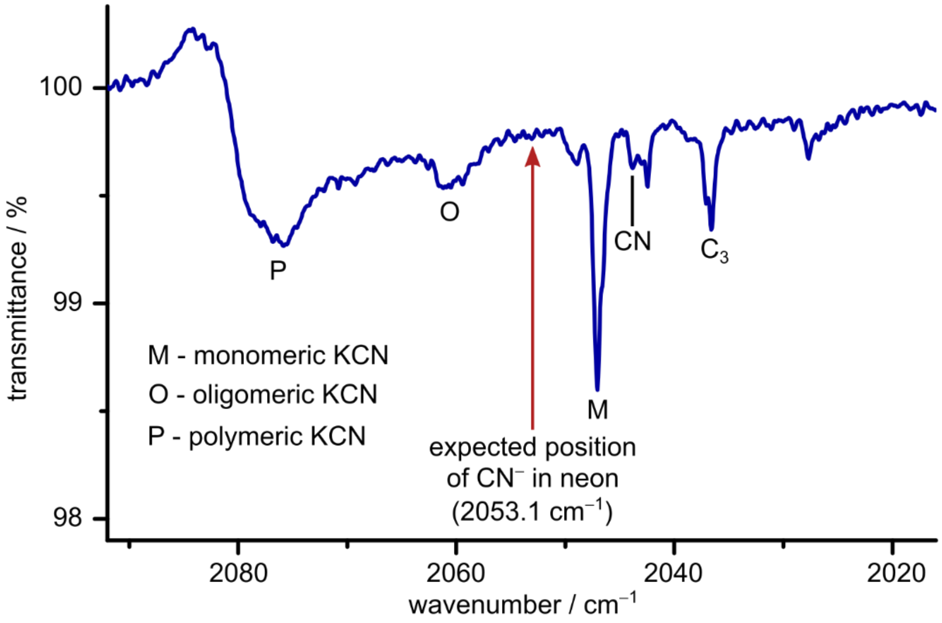

Figure S1. IR spectrum recorded after deposition of laser ablated KCN in excess neon at 6 K showing the CN stretching region and the ν_3 band of the C_3 radical.

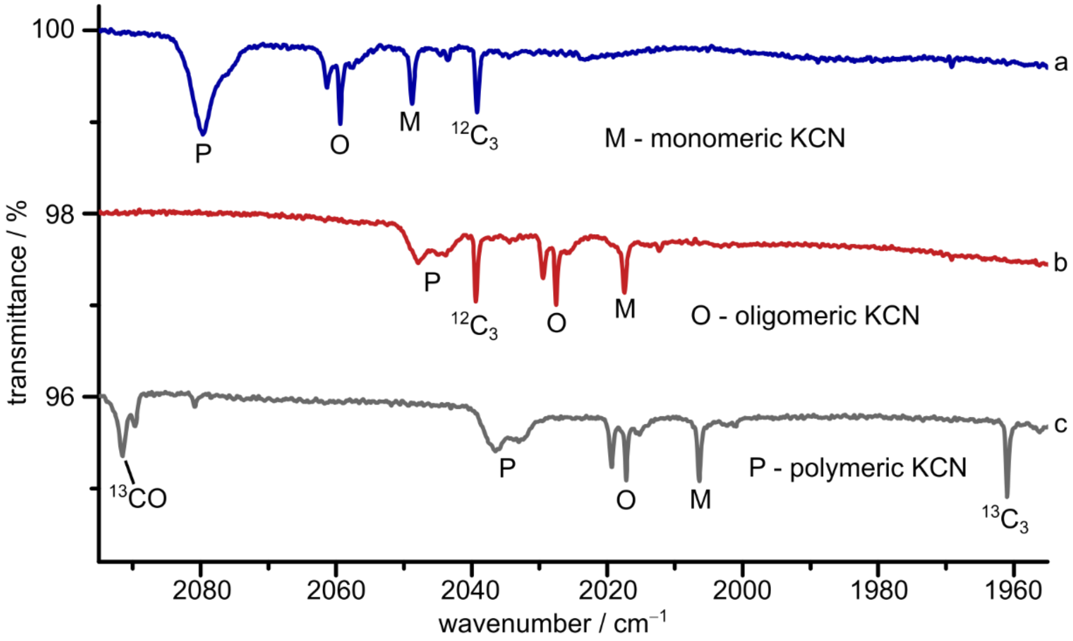

Figure S2. IR spectra recorded after pulsed laser deposition of a) $K^{12}C^{14}N$, b) $K^{12}C^{15}N$, and c) $K^{13}C^{14}N$ in excess argon at 12 K.

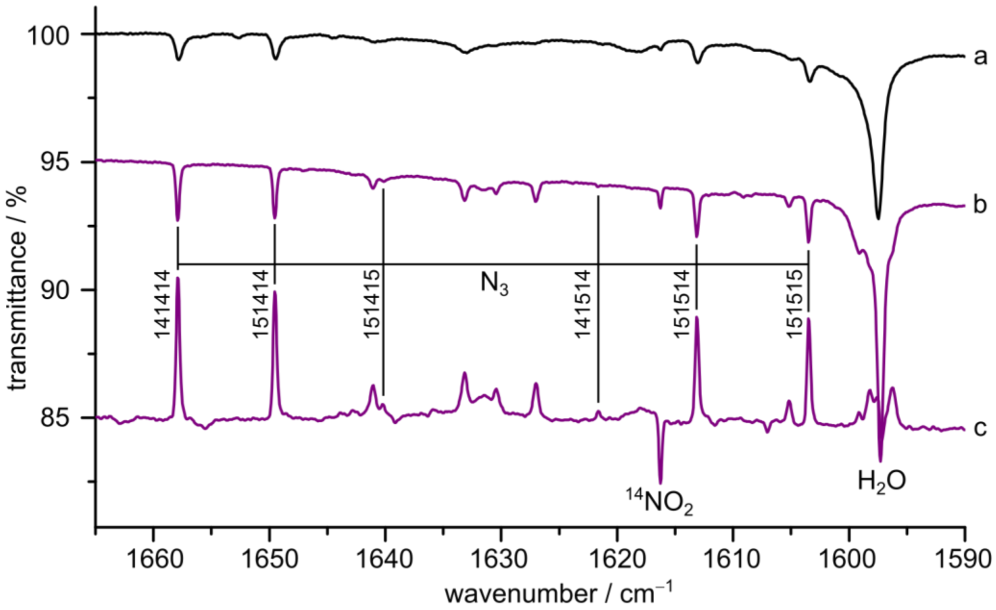

Figure S3. Insert of the IR spectra obtained from reaction products of laser ablated $K^{12}C^{14}N/K^{12}C^{15}N$ (1:1) in solid $^{14}N_2/^{15}N_2$ (1:1) showing the ν_3 region of the N_3 radical and its $^{14/15}N$ isotopologues: a) after deposition at 12 K, b) after annealing to 25 K, and c) difference spectrum after irradiation with UV light (λ = 273 nm).

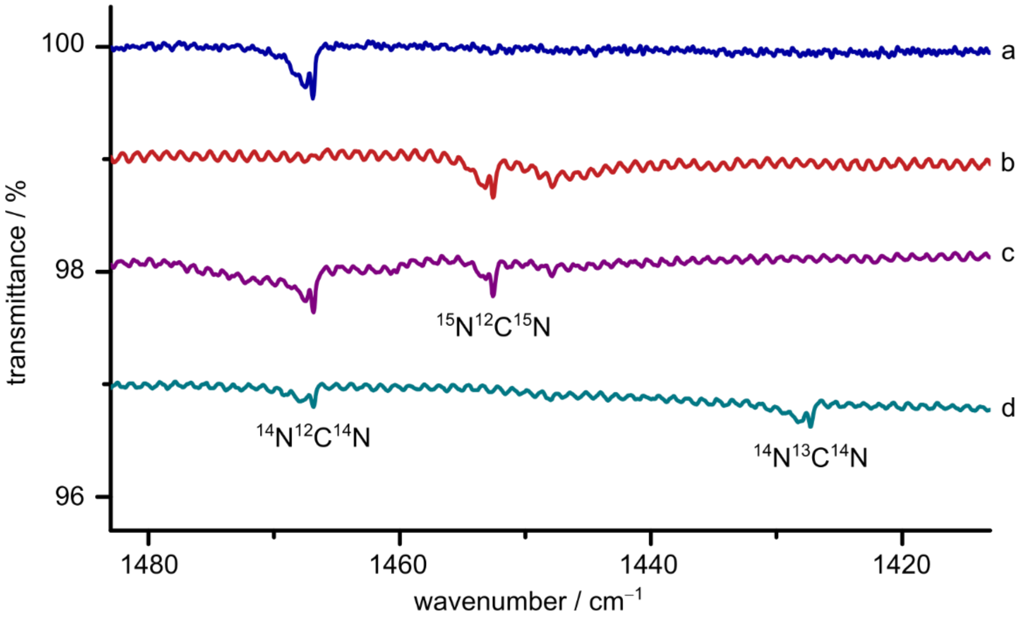

Figure S4. Part of the IR spectra in the ν_3 region of the NCN radical recorded after pulsed laser deposition of KCN in excess neon at 6 K, a) $K^{12}C^{14}N$ with $^{14}N_2$ (0.5 %), b) $K^{12}C^{15}N$ with $^{15}N_2$ (0.5 %), c) $K^{12}C^{14}N/K^{12}C^{15}N$ (1:1) with $^{14}N_2/^{15}N_2$ (1:1, 0.2 %), d) $K^{12}C^{14}N/K^{13}C^{14}N$ (1:1) with $^{14}N_2$ (0.5 %).

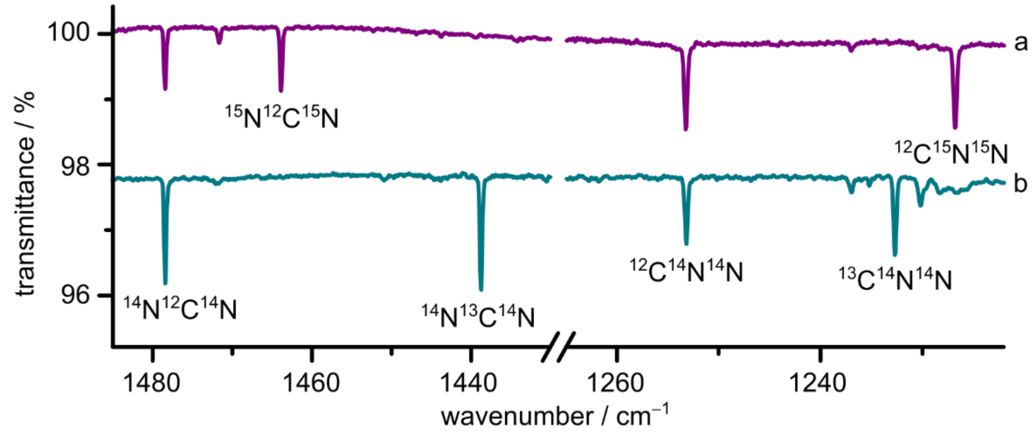

Figure S5. IR spectra recorded after co-deposition of a) $K^{12}C^{14}N/K^{12}C^{15}N$ (1:1) in solid $^{14}N_2/^{15}N_2$ (1:1) and b) $K^{12}C^{14}N/K^{13}C^{14}N$ (1:1) in solid $^{14}N_2$ at 12 K after annealing to 25 K.

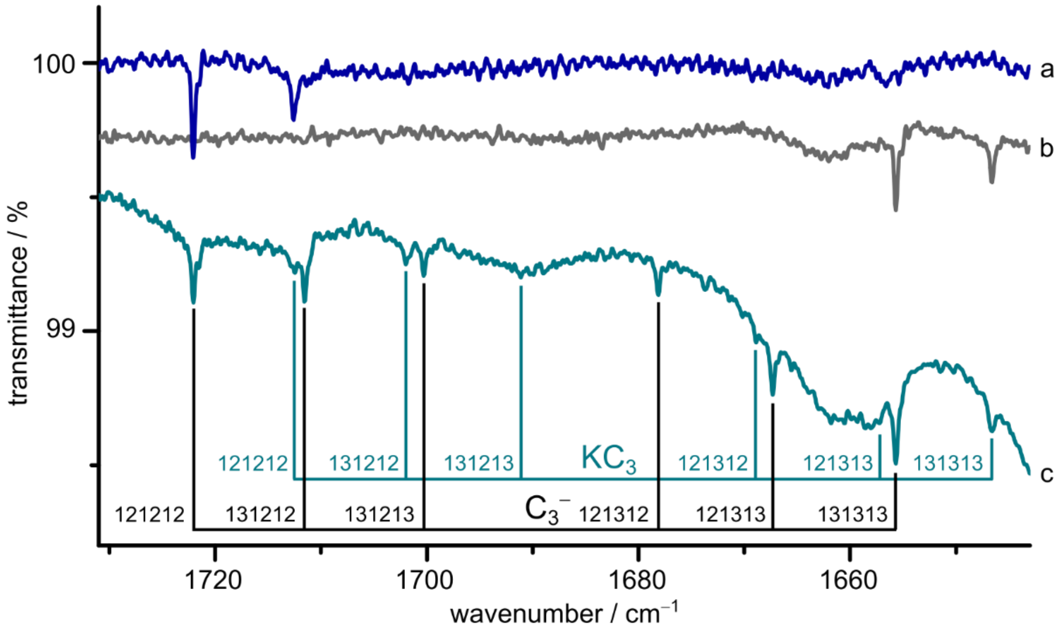

Figure S6. IR spectra in the region of the ν_3 band of the C_3^- ion and its $^{12/13}C$ isotopologues obtained after deposition of laser ablated a) $K^{12}C^{14}N$, b) $K^{13}C^{14}N$, and c) $K^{12}C^{14}N/K^{13}C^{14}N$ 1:1 in excess argon at 12 K.

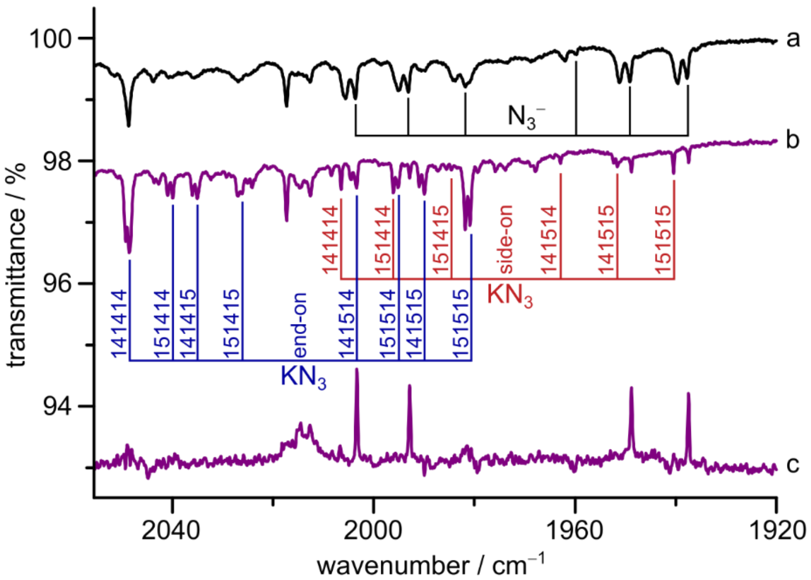

Figure S7. IR spectra in the ν_3 stretching region of N_3^- obtained from reaction products of laser ablated $K^{12}C^{14}N/K^{12}C^{15}N$ (1:1) in solid $^{14}N_2/^{15}N_2$ (1:1) a) after deposition at 12 K, b) after annealing to 25 K, and c) difference spectrum after irradiation with UV light (λ = 273 nm). Bands pointing upwards indicate depletion of the corresponding species.

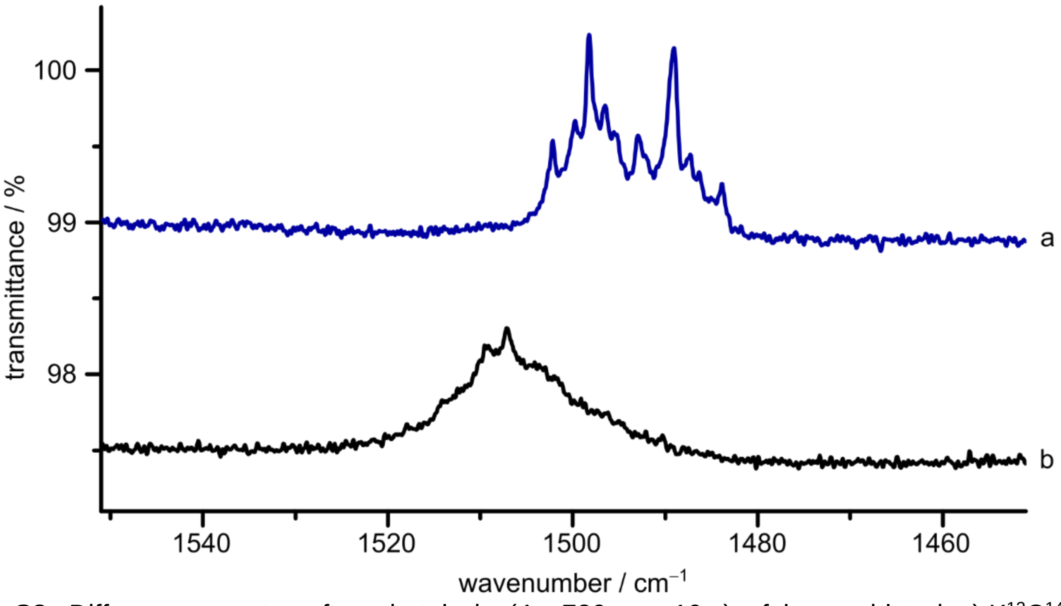

Figure S8. Difference spectra after photolysis (λ = 730 nm, 10 s) of laser ablated a) $K^{12}C^{14}N$ and b) $Na^{12}C^{14}N$ co-deposited with low concentrations of $^{14}N_2$ in excess neon at 6 K. Bands pointing upwards indicate depletion of the corresponding species.

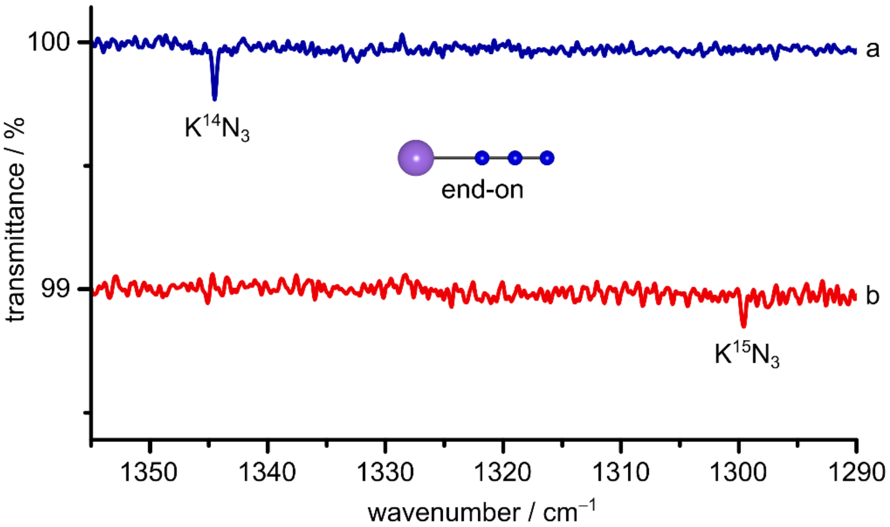

Figure S9. Difference spectra in the ν_1-region of end-on KN_3 after photolysis (λ = 470 nm, 10 min) of laser ablated a) $K^{12}C^{14}N$ co-deposited with traces of $^{14}N_2$ and b) $K^{12}C^{15}N$ co-deposited with $^{15}N_2$ (0.5 %) in excess neon at 6 K. Bands pointing downwards indicate formation of the corresponding species.

S4. Tables of additional experimental IR bands

Table S1. Further experimental IR bands (cm^{-1}) observed after isolation of laser ablated potassium cyanide in solid neon at 6 K.

**KC^{14}N**	**KC^{15}N**	**K^{13}CN**	**Assignment**
2179.1	2164.6	2120.4	KNCO (ν_3)
2075.7	2043.7	2034.8	KCN (polymers)
2061.2	2027.7	2017.0	KCN (oligomers)
2047.0	2015.7	2004.7	KCN (monomer)
2043.8	2012.6	2002.2	CN
2036.6	2036.6	1958.6	C_3 (ν_3)
1466.9	1452.5	1427.2	NCN (ν_3)
**KC^{14}N + $^{14}N_2$**	**KC^{14}N + KC^{15}N + $^{14}N_2$ + $^{15}N_2$**		**Assignment**
	2266.1		$^{14}N^{14}N^{15}N^{15}N^+$ (ν_s)
2237.8	2237.8, 2179.0, 2163.3		N_4^+ (ν_{as})
1498.2, 1489.0	1498.2, 1489.0, 1471.6, 1463.0, 1448.4, 1439.1		?

Table S2. Further experimental IR bands (cm^{-1}) observed after isolation of laser ablated potassium cyanide in solid argon at 12 K.

**KC^{14}N**	**KC^{15}N**	**K^{13}CN**	**Assignment**
2173.7	2158.8	2115.4	KNCO (ν_3)
2079.6	2047.9	2036.4	KCN (polymers)
2061.3, 2059.3	2029.4, 2027.5	2019.3, 2017.2	KCN (oligomers)
2048.7	2017.5	2006.4	KCN (monomer)
2043.5	2012.3	2001.1	CN
2039.2	2039.2	1960.9	C_3 (ν_3)

Table S3. Further experimental IR bands (cm^{-1}) observed after isolation of laser ablated potassium cyanide in solid nitrogen at 12 K.

**KC^{14}N + $^{14}N_2$**	**KC^{14}N + KC^{15}N + $^{14}N_2$ + $^{15}N_2$**	**Assignment**
2328.3	2328.3, 2250.5	N_2
2003.3	2003.3, 1992.8, 1948.7, 1937.4	N_3^- (ν_3)
1657.9	1657.9, 1649.5, 1641.0, 1621.6, 1613.1, 1603.4	N_3 (ν_3)
1575.4	1575.4, 1547.1, 1523.1	?
**KC^{14}N + KC^{15}N + $^{14}N_2$ + $^{15}N_2$**	**K^{12}CN + K^{13}CN + $^{14}N_2$**	
1478.4, 1463.8	1478.4, 1438.7	NCN (ν_3)
1253.1, 1226.7	1253.1, 1232.6	CNN (ν_3)

S5. Calculated structures and harmonic frequencies

KN_3 (C_{2v}), $^1A_1$, CCSD(T)/ma-def2-TZVP

N	0.000000	0.000000	0.074595
N	1.188023	0.000000	0.142970
N	-1.188023	0.000000	0.142970
K	0.000000	0.000000	2.624664

b_1	133	(1)
a_1	255	(61)
b_2	630	(1)
a_1	678	(0)
a_1	1301	(0)
b_1	2039	(613)

KN_3 ($C_{\infty v}$), $^1A_1$, CCSD(T)/ma-def2-TZVP

K	0.000000	0.000000	2.946988
N	0.000000	0.000000	0.540070
N	0.000000	0.000000	-0.661670
N	0.000000	0.000000	-1.825389

π	79	(15)
σ^+	280	(72)
π	550	(6)
	643	(5)
σ^+	1358	(57)
σ^+	2124	(950)

KC_3 (C_{2v}), $^2B_1$, CCSD(T)/ma-def2-TZVP

C	0.000000	0.000000	-0.011005
C	1.304926	0.000000	0.135572
C	1.304926	0.000000	0.135572
K	0.000000	0.000000	2.725064

b_1	142	(3)
b_2	186	(36)
a_1	236	(65)
a_1	440	(4)
a_1	1182	(0)
b_1	1727	(224)

NaC_3 (C_{2v}), $^2B_1$, CCSD(T)/ma-def2-TZVP

C	0.000000	0.000000	0.073560
C	1.304359	0.000000	0.239618
C	-1.304359	0.000000	0.239618
Na	0.000000	0.000000	2.432408

b_1	140	(4)
b_2	148	(36)
a_1	293	(75)
a_1	459	(1)
a_1	1178	(0)
b_1	1712	(223)

S6. References

[1] a) F. Neese, *WIREs Comput. Mol. Sci.* **2012**, *2*, 73; b) F. Neese, *WIREs Comput. Mol. Sci.* **2017**, *2*, e1327.

[2] a) A. D. Becke, *Phys. Rev. A* **1988**, *38*, 3098; b) John P. Perdew, *Phys. Rev. B* **1986**, *33*, 8822.

[3] a) S. Grimme, J. Antony, S. Ehrlich, H. Krieg, *J. Chem. Phys.* **2010**, *132*, 154104; b) S. Grimme, S. Ehrlich, L. Goerigk, *J. Comput. Chem.* **2011**, *32*, 1456.

[4] a) F. Weigend, R. Ahlrichs, *Phys. Chem. Chem. Phys.* **2005**, *7*, 3297; b) J. Zheng, X. Xu, D. G. Truhlar, *Theor. Chem. Acc.* **2011**, *128*, 295.

[5] W. Weltner, P. N. Walsh, C. L. Angell, *J. Chem. Phys.* **1964**, *40*, 1299.

[6] R. Tian, J. C. Facelli, J. Michl, *J. Phys. Chem.* **1988**, *92*, 4073.

[7] F. F. Martinotti, M. J. Welch, A. P. Wolf, *Chem. Commun.* **1968**, 115.

[8] J. Drowart, R. P. Burns, G. DeMaria, M. G. Inghram, *J. Chem. Phys.* **1959**, *31*, 1131.

[9] a) P. Harteck, R. R. Reeves, G. Mannella, *J. Chem. Phys.* **1958**, *29*, 608; b) T. Yamashita, *J. Chem. Phys.* **1979**, *70*, 4248.

[10] D. Forney, W. E. Thompson, M. E. Jacox, *J. Chem. Phys.* **1992**, *97*, 1664.

[11] M. E. Jacox, W. E. Thompson, *J. Chem. Phys.* **2007**, *126*, 54308.

[12] a) R. L. DeKock, W. Weltner, *J. Am. Chem. Soc.* **1971**, *93*, 7106; b) D. E. Milligan, M. E. Jacox, *J. Chem. Phys.* **1966**, *44*, 2850.

[13] N. G. Moll, W. E. Thompson, *J. Chem. Phys.* **1966**, *44*, 2684.

[14] David R. Lide (Editor), *Handbook of chemistry and physics* **1992**.

[15] H. Hotop, W. C. Lineberger, *J. Phys. Chem. Ref. Data* **1985**, *14*, 731.

[16] C.-G. Zhan, J. A. Nichols, D. A. Dixon, *J. Phys. Chem. A* **2003**, *107*, 4184.

[17] R. L. Jackson, M. J. Pellerite, J. I. Brauman, *J. Am. Chem. Soc.* **1981**, *103*, 1802.

[18] M. Zhou, L. Andrews, *J. Phys. Chem. A* **2000**, *104*, 1648.

[19] L. Andrews, M. Zhou, G. V. Chertihin, W. D. Bare, *J. Phys. Chem. A* **2000**, *104*, 1656.

[20] H. A. Papazian, *J. Chem. Phys.* **1961**, *34*, 1614.

[21] W. E. Thompson, M. E. Jacox, *J. Chem. Phys.* **1990**, *93*, 3856.
